# Compensatory Mechanisms Modulate the Neuronal Excitability in a Kainic Acid-Induced Epilepsy Mouse Model

**DOI:** 10.3389/fncir.2018.00048

**Published:** 2018-06-29

**Authors:** Gaojie Pan, Zhicai Chen, Honghua Zheng, Yunwu Zhang, Huaxi Xu, Guojun Bu, Hui Zheng, Yanfang Li

**Affiliations:** ^1^Fujian Provincial Key Laboratory of Neurodegenerative Disease and Aging Research, Institute of Neuroscience, Medical College, Xiamen University, Xiamen, China; ^2^Neurodegenerative Disease Research Program, Sanford-Burnham Medical Research Institute, La Jolla, CA, United States; ^3^Department of Neuroscience, Mayo Clinic, Jacksonville, FL, United States; ^4^Huffington Center on Aging, Baylor College of Medicine, Houston, TX, United States

**Keywords:** epilepsy, kainic acid, hyperexcitability, synaptic transmission, γ-aminobutyric acid, tonic inhibition

## Abstract

Epilepsy is one of the most common neurological disorders affecting millions of people. Due to the complicated and unclear mechanisms of epilepsy, still a significant proportion of epilepsy patients remain poorly controlled. Epilepsy is characterized by convulsive seizures that are caused by increased excitability. In this study, by using kainic acid (KA)-induced epilepsy mice, we investigated the neuronal activities and revealed the neuronal compensatory mechanisms after KA-induced toxic hyperexcitability. The results indicate that both phasic inhibition induced by enhanced inhibitory synaptic activity and tonic inhibition mediated by activated astrocytes participate in the compensatory mechanisms. Compensatory mechanisms were already found in various neuronal disorders and were considered important in protecting nervous system from toxic hyperexcitability. This study hopefully will provide valuable clues in understanding the complex neuronal mechanisms of epilepsy, and exploring potential clinical treatment of the disease.

## Introduction

Epilepsy is one of the most common neurological disorders affecting 1%–3% of the general population worldwide, and with a tendency to increase annually (Sander, 2003; Duncan et al., 2006). It could be caused by multiple reasons including genetic mutations, cerebral trauma, ischemia and high fever. Even though various antiepileptic drugs were applied clinically, still a significant proportion of epilepsy patients remain poorly controlled (Regesta and Tanganelli, 1999; Wiebe and Jette, 2012).

To investigate the underlying mechanisms of epilepsy and explore potential therapeutic treatments, various epilepsy animal models have been developed. Systemic administration of certain convulsant agents is the most common method to create acute models of seizures, among which kainic acid (KA)-induced model is widely used to study the pathogenesis of epilepsy and antiepileptic drugs (Ben-Ari and Cossart, 2000; Laurén et al., 2010; Obeid et al., 2010). KA is an analog of glutamate and agonist to activate ionotropic glutamate receptor, resulting in cation influx and membrane depolarization, eliciting its excitatory effect (Nadler, 1981; Wang et al., 2005).

In vertebrate central nervous system, neuronal excitation is mainly mediated by excitatory neurotransmitters glutamate and acetylcholine, while neuronal inhibition is primarily mediated by γ-aminobutyric acid (GABA). In physiological conditions, neuronal excitation and inhibition in central nervous system maintains a delicate balance, which could be broken in pathological conditions, resulting in neurological disorders such as epilepsy, depression and schizophrenia. Epilepsy is characterized by epileptic seizures, the hypersynchronous electrical discharges in the brain (Isomura et al., 2008; Avoli and de Curtis, 2011). The direct cause of convulsive seizures was thought to result from increased excitability or decreased inhibition in the brain, though the detailed mechanisms are still unclear. In the present study, we observed interesting alteration of neuronal activities in KA-induced epilepsy model mice. Acutely after KA-induced hyperexcitability, there emerged the compensatory mechanisms, which could be considered a protective activity to neutralize the toxic hyperexcitability. Further investigation revealed that in KA-induced epilepsy mice, the compensatory mechanisms include not only enhanced phasic inhibition induced by inhibitory synaptic activity, but also increased tonic inhibition mediated by activated astrocytes. This study might provide important clue in understanding and clarifying the mechanisms of epilepsy.

## Materials and Methods

### Experimental Animals

Adult C57BL/6 mice (2–4-month old) of both sexes were used. This study was carried out in accordance with the recommendations of the National Institutes of Health’s Guidelines for the Care and Use of Laboratory Animals and the protocol was approved by the Animal Ethics Committee of Xiamen University.

### Antibodies and Reagents

The rabbit anti-GAD65/67 antibody was from Millipore (catalog #AB1511). The mouse anti-GFAP (catalog #3670) antibody was from Cell Signaling Technology. The rabbit anti-GAPDH (catalog #ab181602) antibody was from Abcam. The KA was from Sigma (catalog #K0250). The HRP-conjugated secondary antibodies were from Thermo Fisher Scientific and the Alexa Flour-conjugated secondary antibodies were from Invitrogen. The glutamate receptor antagonists CNQX (catalog #C127) and D-AP5 (catalog #A5282) were purchased from Sigma. The picrotoxin (PTX) was from Abcam (catalog #ab120315). All the other reagents used for preparing electrophysiological recording solutions were purchased from Sigma.

### Behavioral Analysis

The mouse was placed in a 20 × 35 × 25 cm cage, filled with some bedding material for comfort. The mouse was intraperitoneally injected with KA solution (15 mg/kg body weight) or saline as control. The mouse was closely observed for about 3 h and the severity of seizure was assessed according to Racine’s scale, which is widely used in rodent models of experimental epilepsy (Racine, 1972; Lüttjohann et al., 2009). Racine’s scale categorizes five grades of seizure intensity based on the animal behavior, grade 1 to grade 5 from modest to the severest. Seizure grade 1 was characterized by the mouth and facial movements of experimental mouse. Head nodding and shaking of mouse was referred as grade 2. The seizure grade 3 was characterized by the mouse forelimb clonus or shaking. Seizure characterized by rearing was referred as grade 4, whereas rearing and falling was referred as grade 5, the strongest intensity of seizures.

### Electroencephalography (EEG) Recording

For electroencephalography (EEG) recording at 30 min and 2 h after KA injection, mouse was anesthetized by intraperitoneal injection of 5% chloral hydrate at a dose of 350 mg/kg body weight. In about 20–30 min, the mouse entered unconscious state, with no response when its toes were pinched. The mouse was then fixed at stereotaxic frame, and the EEG recording system was set up. In about 20 min, the mouse started to recover from deep anesthesia, with eyes blinking when touched. KA solution was then intraperitoneally injected to the mouse at a dose of 15 mg/kg body weight. Right after KA injection, the EEG recording was started to get the EEG data at 30 min and 2 h after KA injection. For EEG recording at 8 h, 24 h and 1 week after KA injection, the mouse was injected with KA first. Then at 1 h before the specific time points, mouse was anesthetized and set up for EEG recording. The recording lasted for 1.5 h to get the EEG data at specific time points. The EEG recording of mouse was performed by using the Cadwell 32 Channel EEG Amplifier (Easy II) and analyzed with Easy EEG Acquire software (Cadwell, Version 2.0.1). For setting up the EEG recording system, the ground electrode was inserted into the muscle at 0.5 cm above the mouth, to connect the mouse to the ground as zero. The reference electrode was inserted at 1 cm above the nose of mouse, to eliminate the internal wave induced by the machine. Two recording electrodes were inserted into the mouse scalp above each eye. Each recording electrode touches three functional positions at the mouse head, therefore six channel whole brain EEG was recorded. Before each recording, the system was tested to make sure the total recording resistance was below 5 kΩ.

### Western Blotting

Briefly, the cortex of mice was dissected and homogenized with RIPA buffer (10 mM Tris-HCl, 137 mM NaCl, 0.1% SDS, 1% Triton X-100, 1% sodium deoxycholate, pH 7.4) containing protease inhibitor. After centrifugation at 12,000 rpm for 20 min, the collected protein samples were subjected to SDS-PAGE. The transferred blots were then incubated with primary antibodies at 4°C overnight and HRP-conjugated secondary antibodies (Thermo Fisher Scientific) at room temperature for 2 h, then detected with enhanced chemiluminescence (Millipore, Cat# WBKLSO500). ImageJ software was applied to determine the intensity of each indicated blotting bands.

### RNA Isolation and Quantitative RT-PCR

Cortical RNA was extracted with TRIzol reagent (Invitrogen, Cat# 15596-018) and immediately reverse transcribed to cDNA by using GoScript Reverse transcription Kit (Promega, Cat# A5001). Quantitative RT-PCR was then conducted with Applied Biosystems (ABI) 7500.

The primers used for mouse gene expressions were as follows:

GFAP forward: 5′-TCCTGGAACAGCAAAACAAG-3′

GFAP reverse: 5′-CAGCCTCAGGTTGGTTTCAT-3′

GAD forward: 5′-TCGGATCTGAAGATGGCTCTGC-3′

GAD reverse: 5′-TAGAGCAGAGCGCACAGCTT-3′

GAPDH forward: 5′-GGTGAAGGTCGGTGTGAACG-3′

GAPDH reverse: 5′-CTCGCTCCTGGAAGATGGTG-3′

### Immunofluorescence of Brain Slices

Anesthetized mice were perfused with 0.9% saline, then 4% paraformaldehyde for fixation. Excised brains were post-fixed overnight in 4% paraformaldehyde, then 30% sucrose at 4°C followed by freezing. Frozen brains were sectioned into 15 μm thick slices. For immunofluorescence, brain slices were sequentially incubated with primary antibodies and secondary antibodies. The images for mounted brain sections were taken with Nikon A1R laser confocal microscope and analyzed with ImageJ program.

### Electrophysiological Recording

Mice were anesthetized and decapitated. The quickly excised brain was cut into 400 μm-thick slices with vibrating microtome in ice cold ACSF (in mM: 126 NaCl, 18 NaHCO_3_, 2.5 KCl, 1.2 NaH_2_PO_4_, 1.2 CaCl_2_, 2.4 MgCl_2_, 11 glucose). The brain slices were incubated at 32°C for 30 min and then room temperature for at least 30 min before use. For whole-cell recording, brain slices were immersed in ACSF with continuous perfusion. During the entire process of brain slice preparation and recording, the ACSF was continuously aerated with 95% O_2_–5% CO_2_. The composition of pipette solution is (in mM: 140 CsCH_3_SO_3_, 2 MgCl_2_, 5 TEA-Cl, 10 HEPES, 1 EGTA, 2.5 Mg-ATP, 0.3 Na_2_-GTP, adjusted to pH 7.2–7.4 with CsOH) for recording of phasic sEPSCs, sIPSCs and tonic inhibition. The sEPSCs were recorded at a holding potential of −70 mV. The sIPSCs and tonic inhibition were recorded at a holding potential of 0 mV. For tonic inhibition recording, 100 μM PTX was used to block GABA_A_ receptors (mGluRs). In addition, 5 μM GABA was added into ACSF to create larger tonic inhibition. For determining the neuronal excitability, the pipette solution contains in mM: 140 K-gluconate, 2 MgCl_2_, 0.1 CaCl_2_, 10 HEPES, 1.1 EGTA, 0.3 Na_2_-GTP, Na_2_-ATP, adjusted to pH 7.25 with KOH. The action potential was evoked with different intensity of injected currents.

Along with recording, raw data were collected with Multi-Clamp 700B amplifier (Molecular Devices) filtered at 1 kHz, sampled and digitized with Digidata 1440A (Molecular Devices). Recording protocols were set with Clampex 10 acquisition software (Molecular Devices). Data were analyzed by using pClamp10.3 software and Mini analysis software.

### Statistical Analyses

The data were analyzed by using Microsoft Excel or GraphPad Prism program and presented as mean ± SEM. Student’s *t*-test was applied to compare the difference between two groups. For comparison of the current-evoked spikes between control and KA-injected mice, two-way ANOVA was used. *P* values less than 0.05 were considered statistically significant (*P < 0.05, **P < 0.01, ***P < 0.001).

## Results

### Induction of Seizures in Mice With Kainic Acid (KA) Administration

Adult mice of 2–4-months old were intraperitoneally injected with KA solution at the dose of 15 mg/kg body weight or saline as control. The behavior responses of the mice were closely observed and their intensities of seizure were assessed according to Racine’s scale, which is a widely used tool in rodent animal models of experimental epilepsy (Racine, 1972; Lüttjohann et al., 2009). All examined mice injected with KA showed clear onset of epileptic behavior within 15 min and lasted for 30 min to 2 h. The intensity of KA-induced seizures was analyzed based on Racine’s scale (Table 1). Most of the KA-injected mice developed seizures of grade 3 (30%) or grade 4 (35%), characterized by apparent forelimb clonus and rearing. The behavioral assessment result indicates that the KA-induced epilepsy mouse model was successfully developed and could be used for later study.

**Table 1:** The evaluation of kainic acid (KA)-induced epilepsy.

Seizure severity	Mice number	Onset of convulsions (s)
Grade I	0	N/A
Grade II	7	649.6 ± 44.8
Grade III	12	246.8 ± 53.8
Grade IV	14	337.8 ± 52.9
Grade V	7	370.3 ± 90.4

### The Acute and Chronic Changes of Neuronal Excitability After KA Injection

**Figure 1:**
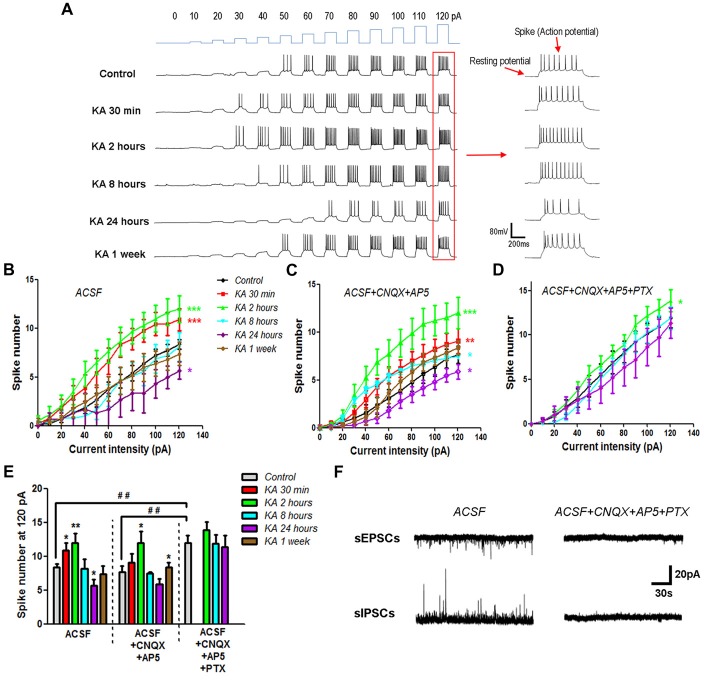
The neuronal excitability changed after kainic acid (KA) injection. **(A)** On the cortex of mice brain slices, depolarization currents with various intensities were injected into individual neurons to induce excitatory effect. Representative traces of the resting membrane potential (RP) baseline and evoked action potentials (spikes). **(B)** Evoked action potentials were quantified to compare the neuronal excitability at different time points after KA injection (*n* = 10–13 cells from 3–4 mice in each group). **(C)** Action potentials were evoked by depolarization currents and quantified in the presence of 20 μM CNQX and 50 μM AP5 (*n* = 11–13 cells from three mice in each group). **(D)** Action potentials evoked and quantified in the presence of 20 μM CNQX, 50 μM AP5 and 100 μM PTX (*n* = 9–13 cells from two mice in each group). **(E)** The number of spikes stimulated by 120 pA depolarization current was quantified in different conditions. *P < 0.05, **P < 0.01, ***P < 0.001 compared to control in the same recording condition. ^##^*P* < 0.01 of control mice compared between different conditions. **(F)** Spontaneous excitatory (sEPSCs) and inhibitory (sIPSCs) postsynaptic currents were recorded in the absence or presence of CNQX, AP5 and PTX.

The neuronal excitability change in KA-induced epilepsy mice was determined by using current-clamp recording. Cortical pyramidal neurons in the mice brain slices were injected with programed currents of specific intensity. The positively charged currents could induce cell membrane depolarization and trigger action potentials when depolarization reaches the threshold (Figure 1A). By quantifying the number of evoked action potentials (spikes), the neuronal excitability change after KA injection was determined. The more action potentials is triggered, the higher is the neuronal excitability. Compared to control mice that injected with normal saline (0.9% NaCl), the general neuronal excitability increased immediately after KA injection, and reached the peak at 2 h, then reduced gradually afterward. At 8 h after KA injection, the neuronal excitability was reduced to the similar level of control mice, and decreased continuously. At 24 h after KA injection, the neuronal excitability was significantly lower than control. The chronic effect of KA on neuronal excitability was also investigated. One week after KA injection, the general neuronal excitability went back to normal level (Figures 1B,E). These results indicate that after KA administration, cortical neuronal excitability underwent an oscillating process. It increased rapidly at the beginning, then decreased gradually, eventually was restored back to the normal level.

It’s known that KA is an analog of glutamate and activates the ionotropic glutamate receptor by itself. In order to examine whether the neuronal excitability change after KA injection is due to the increase of ambient glutamate concentration, electrophysiological recording was performed in the presence of 20 μM CNQX and 50 μM AP5, the specific antagonists of AMPA and NMDA type glutamate receptors, respectively. Similar neuronal excitability change was obtained after the glutamate receptor activity was blocked (Figures 1C,E), which clearly exclude the role of ambient glutamate in the neuronal excitability change of KA-induced epilepsy mouse model. By adding 100 μM PTX, which is the antagonist of inhibitory GABA_A_ receptors, together with CNQX and AP5, we further examined the effect of inhibitory receptors on KA-induced excitability change. Interestingly, the neuronal excitability in control mice injected with normal saline increased significantly in the presence of PTX, CNQX and AP5. However, the excitability enhanced only at 2 h, but not 8 h after KA administration. In addition, at 24 h after KA injection, the neuronal excitability was no longer significantly different from control mice (Figures 1D,E). This result indicates that inhibitory receptors play critical roles in regulating neuronal excitability after KA injection. The blockade effect of CNQX, AP5 and PTX was also approved with electrophysiological recording. Both spontaneous excitatory (sEPSCs) and inhibitory (sIPSCs) postsynaptic currents were blocked almost completely in the presence of 20 μM CNQX, 50 μM AP5 and 100 μM PTX (Figure 1F).

### Comparison of Resting Membrane Potential (RP) and Membrane Resistance (Rm) of Neurons Recorded After KA Administration

Neuronal excitability could also result from the changes in resting membrane potential (RP) of neurons. As shown in Figure 1A, the RP value could be measured in electrophysiological recording when no current stimulus was applied. To examine this possibility, the RP values of neurons recorded after KA injection (Figures 1A,B) were listed in Table 2. Compared to control, there is no significant difference in RP values at various time points after KA administration. During electrophysiological recording, the membrane resistance (Rm) after whole-cell status development is another important parameter to represent the ion channels’ activity of neurons. So the Rm values of recorded neurons after KA injection were also compared (Table 3), and no significant difference was observed. These results indicate that the neuronal excitability change in KA-induced epilepsy mice is not due to RP change or the differently activated ion channels in recorded neurons.

**Table 2:** The resting membrane potential (RP) of neurons recorded in KA-induced epilepsy mouse model.

	Control (mV)	KA 30 min (mV)	KA 2 h (mV)	KA 8 h (mV)	KA 24 h (mV)	KA 1 week (mV)
#1	−71.18	−68.77	−63.87	−68.08	−59.72	−55.78
#2	−75.85	−72.82	−54.20	−69.64	−60.53	−63.52
#3	−70.86	−54.23	−64.19	−75.91	−58.79	−66.16
#4	−73.28	−73.92	−70.06	−72.63	−69.67	−65.46
#5	−57.98	−79.90	−52.70	−68.12	−72.95	−61.68
#6	−75.63	−69.27	−75.46	−61.23	−71.68	−75.33
#7	−62.66	−73.46	−72.68	−70.81	−77.06	−63.66
#8	−65.52	−66.28	−64.92	−65.38	−78.82	−64.29
#9	−73.51	−59.31	−67.82	−66.25	−66.56	−72.15
#10	−66.32	−70.30	−68.10	−71.35	−67.32	−60.86
#11	−58.18	−72.11	−75.39			−59.23
#12	−62.32		−59.85			
#13	−64.36					
Statistics	−67.51 ± 1.74	−69.12 ± 2.15	−65.77 ± 2.15	−68.94 ± 1.30	−68.31 ± 2.24	−64.37 ± 1.67

**Table 3:** The membrane resistance (Rm) of neurons recorded in KA-induced epilepsy mouse model.

	Control (MΩ)	KA 30 min (MΩ)	KA 2 h (MΩ)	KA 8 h (MΩ)	KA 24 h (MΩ)	KA 1 week (MΩ)
#1	234.1	256.1	326.5	305.8	327.2	212.6
#2	265.1	192.6	264.3	278.5	198.1	258.7
#3	201.7	323.4	286.2	281.9	276.1	230.1
#4	256.2	228.3	241.3	295.4	256.2	183.2
#5	308.6	264.3	255.1	231.1	281.9	269.6
#6	195.0	281.4	298.7	186.9	232.6	221.4
#7	221.4	255.9	273.1	271.2	221.3	257.7
#8	240.9	181.3	331.6	229.8	204.0	206.6
#9	282.5	272.3	316.3	285.6	248.2	243.1
#10	304.6	215.6	252.1	227.9	312.5	315.1
#11	288.3	220.1	210.3			223.6
#12	245.6		238.6			
#13	218.3					
Statistics	250.9 ± 10.4	244.7 ± 12.6	274.5 ± 11.0	259.4 ± 12.1	255.8 ± 13.8	238.3 ± 10.9

### The EEG Recording in KA-Induced Epilepsy Mice

**Figure 2:**
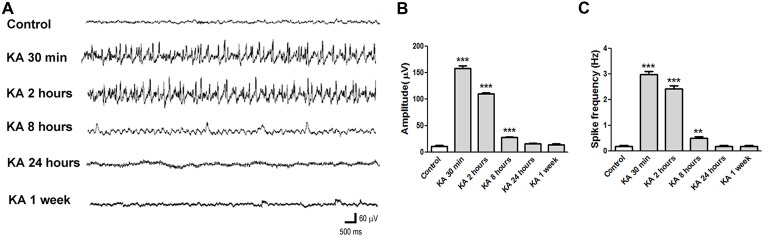
Electroencephalography (EEG) recorded in KA-injected mice. **(A)** Representative EEG traces recorded at different time points after KA injection. **(B,C)** The average amplitude and frequency of EEG waves were quantified (*n* = 3 mice in each group). **P < 0.01, ***P < 0.001.

EEG is a wildly used method to monitor and diagnose epileptic activities of the brain. The EEG recording in KA-injected mice shows consistent result (Figure 2). At 30 min after KA application, high amplitude and high frequency epileptic spikes were recorded and lasted for hours. At 2 h after KA application, even the acute seizure behavior usually has ceased, high amplitude and high frequency ictal like epileptic EEG was still recorded, indicating the increased neuronal excitability. Then at 8 h, 24 h or 1 week after KA injection, the recorded EEG was similar to control, with no obvious epileptic spikes.

### The Changes of Excitatory and Inhibitory Synaptic Function in KA-Induced Epileptic Mice

**Figure 3:**
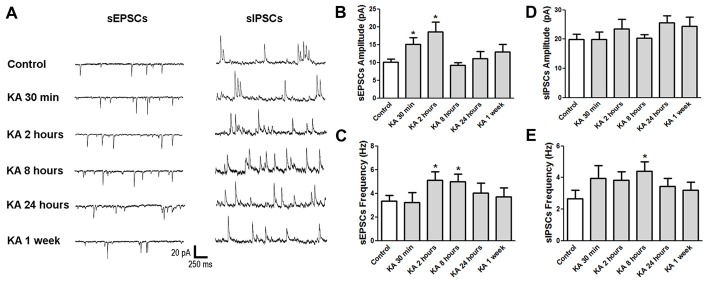
The neuronal synaptic function changed after KA injection. **(A)** Representative traces of sEPSCs and sIPSCs recorded at different time points after KA injection. **(B–E)** The average amplitude and frequency of sEPSCs and sIPSCs were quantified (*n* = 11–17 cells from 3–5 mice in each group). *P < 0.05.

Basically, the neuronal excitability is determined by the balance between excitatory and inhibitory synaptic activities. To investigate the underlying mechanisms of neuronal excitability change after KA-induced epilepsy, mice were sacrificed at various time points after KA administration and the brain slices were subjected to electrophysiological patch-clamp recording. Spontaneous excitatory postsynaptic currents (sEPSCs) and spontaneous inhibitory postsynaptic currents (sIPSCs) were recorded in cortical neurons, respectively and analyzed (Figure 3). At the initial 30 min after KA injection, the sEPSCs amplitude increased significantly, and stayed at a high level at 2 h, with the accompaniment of increased sEPSCs frequency, consistent with the higher excitability at 30 min and 2 h after KA administration. Then the sEPSCs amplitude went down to the normal level as control when measured at 24 h after KA application. Similar to sEPSCs, the sIPSCs frequency also increased until 8 h after KA injection, then went down to normal level. These results indicate that both excitatory and inhibitory synaptic function were enhanced acutely after KA injection, then went to normal chronically.

### Tonic Inhibition Was Enhanced at 24 h After KA Injection

**Figure 4:**
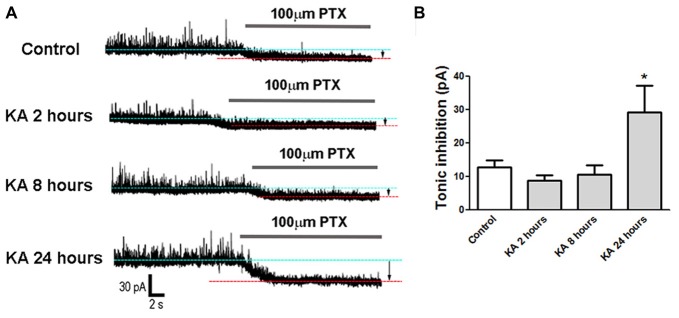
Tonic inhibition recorded in KA-injected mice. **(A)** Tonic inhibitory currents were recorded in mice brain slices at 2 h, 8 h and 24 h after KA injection or control. The gap between two baselines in the absence (blue dotted line) or presence (red dotted line) of 100 μM picrotoxin (PTX) represents tonic inhibition (arrows). **(B)** Tonic inhibition was quantified in control and KA-injected mice (*n* = 7–11 cells from three mice in each group). *P < 0.05.

Besides the phasic synaptic activities, tonic inhibition also contributes to the neuronal excitability. To explain why the neuronal excitability decreased significantly at 24 h after KA injection (Figure 1), patch-clamp was performed to record tonic GABAergic inhibitory currents in cortical neurons. Compared to control mice injected with normal saline, the tonic inhibitory currents increased significantly in KA-administrated mice, indicating an enhanced tonic inhibition 24 h after KA injection. In contrast, the tonic inhibition recorded at 2 h or 8 h after KA treatment didn’t show significant difference from control (Figure 4). This result is consistent with the excitability changes observed in Figure 1 and well explains the reason of decreased excitability at 24 h after KA-induced epilepsy.

### Enhanced Expression Levels of GAD and Reactive Astrocytes After KA Administration

**Figure 5:**
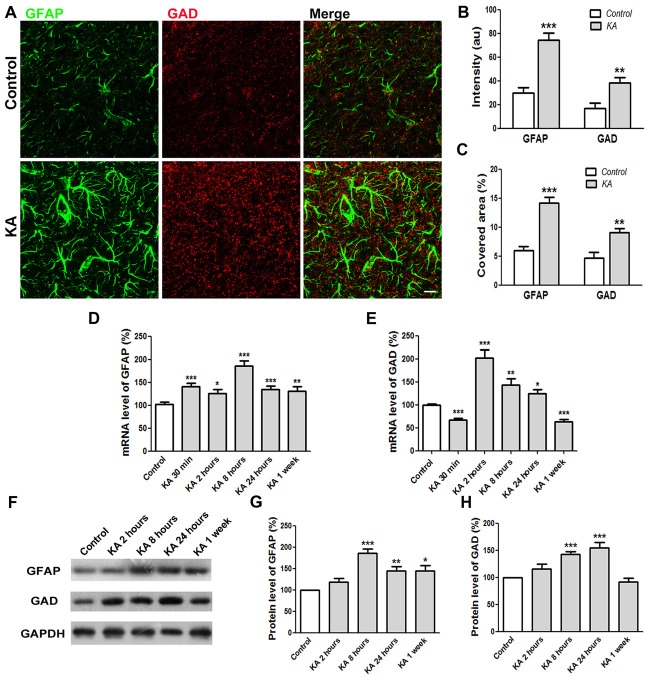
The expression levels of GFAP and GAD increased 24 h after KA injection. **(A)** Co-immunostaining of GFAP (green) and GAD (red) in the cortex of saline-injected control or KA-injected mice at 24 h after injection. **(B)** The quantification of immunofluorescence intensity (*n* = 12 slices from three mice). **(C)** Quantification of immunostaining signal covered area (*n* = 12 slices from three mice). **(D,E)** The mRNA levels of GFAP and GAD were determined by qRT-PCR 24 h after saline or KA injection (*n* = 9). **(F)** Western blot analysis to determine the protein levels of GFAP and GAD in the cortex of control or KA-injected mice. **(G,H)** Quantification of GFAP and GAD protein level changes after KA injection (*n* = 6–8). Scale bar = 10 μm. *P < 0.05, **P < 0.01, ***P < 0.001.

Tonic inhibition was mediated by extrasynaptic GABA_A_ receptors responding to ambient GABA released mainly from reactive astrocytes. To better understand the tonic inhibition change in KA-injected mice brain, immunocytochemistry was performed to determine the expression levels of GAD, the critical enzyme for GABA synthesis, and GFAP, the marker of reactive astrocytes. As shown in Figures 5A–C, 24 h after KA administration, the immunostaining intensity of both GAD and GFAP increased significantly compared to control. Consistently, the covered area of GAD and GFAP immunoreactivity also increased significantly, indicating enhanced GABA release from reactive astrocytes after KA administration. The mRNA levels of GAD and GFAP in the cortex of mice brain were also determined by qRT-PCR. The quantification data show that both mRNA levels were enhanced significantly right after KA injection until 24 h. For chronic effect at 1 week after KA administration, the mRNA level of GFAP still kept at a high level whereas the level of GAD dropped down significantly (Figures 5D,E). Similarly, the protein levels of GFAP and GAD increased significantly acutely after KA administration. At 1 week, the protein level of GFAP, but not GAD, was still significantly higher than control (Figures 5F–H). These results clearly indicate that the expression levels of GFAP and GAD increased after KA injection, at least acutely, which is consistent with the KA-induced increase of tonic inhibition (Figure 4).

## Discussion

In the nervous system, it’s critical to maintain a proper balance between excitatory and inhibitory neuronal activities. The disturbance of this balance results in neurological disorders such as epilepsy, depression and schizophrenia (Kehrer et al., 2008; Cummings et al., 2009; Sun et al., 2009). Epilepsy is a common neurological disorder that affects approximately 50 million people in the world. Although various anticonvulsant drugs have been developed, still about 30% of epilepsy patients are refractory to treatments due to the complex causes and mechanisms of epilepsy (Kwan and Brodie, 2000; Brodie, 2005; Li and Yang, 2017; Lin et al., 2018). In this study, we used KA-induced epilepsy model mice to investigate the acute and chronic changes of neuronal activities and the possible underlying mechanisms.

KA is an agonist to activate glutamate receptor, the major excitatory neurotransmitter receptor in CNS, inducing immediate increase of neuronal excitability (Nadler, 1981; Wang et al., 2005). In this study, we observed hyperexcitability right after KA injection, and significant decrease of neuronal excitability afterward, which could be considered as a compensatory mechanism to counter the toxic hyperexcitability. We then investigated the underlying mechanisms of this neuronal excitability change after KA injection by using glutamate receptor antagonists CNQX and AP5, as well as GABA_A_ receptor antagonist PTX. It seems that blockade of glutamate receptors didn’t change the effect of KA, suggesting that direct activation of ionotropic glutamate receptors solely might not explain the excitability change after KA administration.

It makes us think what mechanisms lead to the hyperexcitability induced by KA. It has been reported that right following KA-induced epilepsy, hippocampal neurons strongly expressed immediate early genes c-Fos and c-Jun, which encode transcription factors (Kim et al., 2008). In the brain of KA model, a bunch of neurotransmitters, receptors and ion channels were observed increased including voltage-gated sodium channel Nav1.6 (Zhu et al., 2016), Na^+^/K^+^/Cl^−^ cotransporter (Nogueira et al., 2015), metabotropic glutamate receptors (mGluRs) especially mGluR2/3 (Caulder et al., 2014) and mGluR5 (Medina-Ceja and García-Barba, 2017), ATP-activated P2Y receptors (Alves et al., 2017) and acetylcholine neurotransmission (Soares et al., 2017). Besides the activation of these excitatory mechanisms, other mechanisms involved in KA-induced hyperexcitability have also been documented. KA receptor activation induced the inhibitory glycine receptor endocytosis (Sun et al., 2014). KA-treated microglia could release tumor necrosis factor alpha (TNFα), leading to increased Ca^2+^ current, more excitatory spikes and further neuronal apoptosis (Zhu et al., 2010). Gap junction that promotes synchronous firing of neurons was also suggested to play an important role in the generation of seizures. Its constructive protein Cx36 was found significantly increased hours after KA administration, then decreased days later (Wu et al., 2017). The accumulating evidence indicates that not only glutamate receptors, but also other various mechanisms were involved in KA-induced hyperexcitability and epileptogenesis.

In the current study, interestingly, by blocking the activity of inhibitory GABA_A_ receptors, the excitability of control mice increased significantly. Compared to this already elevated excitability of control mice, slight but still significant increase of neuronal excitability was only observed at 2 h after KA injection. The excitability decrease at 24 h also disappeared in the presence of PTX. These results indicate that inhibitory GABAergic neurotransmission might play critical roles in regulating the excitability change induced by KA, which could be considered as a compensatory mechanism to protect system from KA-induced hyperexcitability. Indeed, several studies have implied the importance of GABA_A_ receptors and GABAergic neurontransmission system in epileptogenesis (Esclapez and Houser, 1999; Baran et al., 2004; Freichel et al., 2006; González and Brooks-Kayal, 2011).

To better understand the compensatory mechanism, patch-clamp electrophysiological recording was performed in mice brain slices. Results demonstrated that both excitatory and inhibitory synaptic function, as recorded by sEPSCs and sIPSCs of cortical neurons, was enhanced acutely after KA administration. The increase of inhibitory synaptic function (sIPSCs) seems to participate in the compensatory mechanism. However, this result might not explain the significant excitability decrease at 24 h after KA injection. It makes us think about other mechanisms including the role of tonic inhibition. As expected, we found the tonic inhibition increase significantly at 24 h, but not at 2 h or 8 h after KA administration. Further investigation revealed significant increase of expression levels of both GFAP and GAD, confirming the enhanced tonic inhibition mediated by activated astrocytes 24 h after KA injection.

To protect neurons from toxic hyperexcitability, nervous system seems to trigger compensatory enhancement of neuronal inhibitory signaling pathways, therefore neutralize the hyperexcitability back to normal level (Palop et al., 2007). In vertebrate, the neuronal inhibition of central nervous system results from two different sources: phasic inhibition and tonic inhibition (Farrant and Nusser, 2005; McQuail et al., 2015). Phasic inhibition is induced by the presynaptic release of inhibitory neurotransmitter GABA from interneurons. The released GABA activates mainly postsynaptic GABA_A_ receptors, generating synaptic activity dependent phasic inhibition. Phasic inhibition has long been accepted as the most important way to regulate neuronal excitability in CNS. However, besides inhibitory interneurons, astrocytes were also able to release GABA through bestrophin 1 channel when activated pathologically. Released ambient GABA then binds mainly to GABA_A_ receptors at extrasynaptic area in adjacent neurons, causing a sustained tonic inhibition (Glykys and Mody, 2007; Yoon et al., 2012; Lenkov et al., 2013; Yoon and Lee, 2014). Tonic inhibition is important for regulating neuronal activity and neuronal circuit dynamics in both physiological and pathological conditions. Recently, accumulating evidence indicates that tonic inhibition undergoes pathological changes in various neurological disorders including Huntington’s disease, schizophrenia, brain ischemia and Alzheimer’s disease (Brickley and Mody, 2012; Pavlov and Walker, 2013; McQuail et al., 2015; Lin et al., 2018; Rosas-Arellano et al., 2018).

As for the disorder of epilepsy, several studies have observed preserved or enhanced tonic inhibition and demonstrated its regulatory effect on neuronal excitability in various animal models of epilepsy (Li et al., 2013; Pavlov and Walker, 2013). In addition, multiple polymorphisms and mutations in genes encoding extrasynaptic GABA_A_ receptors were found to associate with human epilepsy, emphasizing the potential importance of tonic inhibition in epileptogenesis (Dibbens et al., 2004; Feng et al., 2006; Eugène et al., 2007; Pavlov and Walker, 2013). Tonic inhibition which mediated by extrasynaptic GABA_A_ receptors provides a powerful way to keep the balance of neuronal excitability, therefore has been considered an attractive target for antiepileptic drugs (Li et al., 2013).

Changes in the subunit combination and expression level of GABA_A_ receptors were observed following epilepsy. Unlike synaptic GABA_A_ receptors that mainly combined by α1β3γ2 subunits, extrasynaptic GABA_A_ receptors usually contain δ or ɛ subunit associated with α4, α5 or α6 subunit. This specific subunit combination therefore contributes to their distinct pharmacological features, such as high GABA binding affinity and slow desensitization property (Egawa and Fukuda, 2013; Li et al., 2013; Pavlov and Walker, 2013; van Luijtelaar et al., 2014). In animal models and humans with temporal lobe epilepsy, the expression level of δ subunit was found unexpectedly decreased, while accompanied by increased levels of α4 or α5 subunit (Zhang et al., 2007; Goodkin et al., 2008). The distinct changes of extrasynaptic GABA_A_ receptors confirmed the important regulatory role of tonic inhibition in the disorder of epilepsy.

This current study demonstrated that in KA-induced epilepsy mice, acutely after KA-induced hyperexcitability, compensatory mechanisms including both phasic and tonic inhibitory mechanisms play important roles in regulating neuronal activity back to normal level. The compensatory mechanisms involve the cooperative participation of neurons and activated astrocytes, protecting nervous system from the harmful impairment of toxic epileptic hyperexcitability. This study hopefully will provide valuable clues in understanding the complex neuronal mechanisms of epilepsy, and exploring potential clinical treatment of the disease.

## Author Contributions

YL designed the research and wrote the article. GP and ZC performed the research and analyzed the data. HoZ, YZ, HX, GB and HuZ helped with the data interpretation and discussion.

## Conflict of Interest Statement

The authors declare that the research was conducted in the absence of any commercial or financial relationships that could be construed as a potential conflict of interest.
